# Herbarium collection of the Pontificia Universidad Católica de Valparaíso (PUCV), Chile

**DOI:** 10.3897/BDJ.10.e90591

**Published:** 2022-09-29

**Authors:** Sebastián Cordero, Manuel López-Aliste, Francisca Gálvez, Francisco E. Fontúrbel

**Affiliations:** 1 Pontificia Universidad Católica de Valparaíso, Valparaíso, Chile Pontificia Universidad Católica de Valparaíso Valparaíso Chile

**Keywords:** biological collections, Chile, herbarium specimens, long-term data collections, museum collections

## Abstract

**Background:**

This database gathers 10,721 specimens, belonging to 2,578 species from the Chilean vascular flora (angiosperms, gymnosperms and pteridophytes) deposited in the Herbarium of the Pontificia Universidad Católica de Valparaíso (PUCV) in Chile. The PUCV botanical collection was started by the renowned botanist Otto Zöllner and represents a major natural historical legacy for central Chile, with decades of information represented through preserved specimens. This collection is currently deposited in the Curauma campus of the PUCV. This digitisation effort is part of the PUCV's endeavour to mobilise its biological collections and make them freely available through GBIF, encouraging national and international researchers to generate new knowledge, based on this invaluable heritage, which is a silent witness of the vast plant diversity that once existed in Chile and that is now vanishing due to anthropogenic drivers.

**New information:**

The database provides occurrence records from 10,721 specimens of vascular flora held in the PUCV Herbarium, representing 2,578 species, 914 genera and 177 families. Each record includes data on taxonomy, geographic distribution, elevation and collection information (e.g. date of collection, *legitimavit* and *determinavit* of specimens, general observations). The database serves as a repository containing records from past decades on the diversity and distribution of plant species, mainly from the Chilean Mediterranean biodiversity hotspot.

## Introduction

During the first half of the 20^th^ Century, renowned naturalists were based in central Chile and promoted the creation of the largest and most important biological collections in this South American country. Those collections are the silent witness of the country biodiversity and allow us to reconstruct past species distribution, assess local extinctions and track the spread of invasive species (e.g. Castro et al. 2005, Fuentes et al. 2007, Fuentes et al. 2012). This is the case of the Herbarium of the Pontificia Universidad Católica de Valparaíso (PUCV), which was started by the German botanist Otto Zöllner. Dr. Zöllner began this collection from scratch and also made a great contribution to the training of local experts in plant taxonomy, which were particularly scarce in Chile at that time. During several decades, Dr. Zöllner and his collaborators, along with many students, studied the flora of central Chile, which is considered a biodiversity hotspot due to the high proportion of endemic species (Letelier et al. 2020, Mittermeier et al. 2011) and the specialised ecological interactions of its flora with pollinator species (Medel et al. 2018). Despite its importance, the flora of central Chile is highly threatened by urban encroachment and the expansion of agricultural lands (mainly avocado farms, Armesto et al. 2010). Thus, the PUCV Herbarium comprises a representative sample of central Chile flora prior to the rapid degradation that began in the decade of 1990. The specimens preserved in the PUCV Herbarium constitute a priceless baseline of the biodiversity that once existed in Chile and now cannot be found due to land-use changes (Miranda et al. 2016).

The digitisation of the Herbarium is part of an institutional endeavour to recover and modernise the biological collections held within the University, as they are an important natural heritage. Following the guidelines of the Chilean Ministry of Environment, the first step in this direction is to digitise and mobilise collection specimens to digital platforms, such as the Global Biodiversity Information Facility (GBIF). Thus, the database presented here was developed taking the FAIR (Findable, Accessible, Interoperable and Reusable) principles in mind (Wilkinson et al. 2016). The next step would be the modernisation of the collection, taking advantage of modern technologies that allow inclusion of additional related information besides the preserved species following the extended specimen concept (Lendemer et al. 2019), which expands the possibilities of research and education that can be achieved with biological collections by including extra information that is usually lost after specimen preservation (e.g. tissue samples for DNA extraction, chemical profiles and ecological context information; Teixeira-Costa et al. 2022).

## General description

### Purpose

The main aim is to mobilise the PUCV biological collections, making them freely available while encouraging researchers to generate new knowledge, based on this invaluable heritage.

## Project description

### Title

Vascular flora from the PUCV Herbarium.

### Study area description

Chile, South America.

## Sampling methods

### Study extent

This database contains the specimens of vascular flora recorded in the Herbarium collection held at the Pontificia Universidad Catolica de Valparaíso, indexed on Index Herbariorum as PUCV. The collection results from almost a century of work initiated by Prof. Otto Zöllner and continued by many professors and students.

### Sampling description

Specimen processing follows standard procedures for fresh material collection and dried material mounting and conservation (Bridson and Forman 2000). Specimens are collected from the field and pressed and dried at the laboratory. Then, dried specimens are mounted on acid-free paper using cloth tape and labelled, providing information on taxonomy, geographic distribution, altitude, collection date, *legitimavit* and *determinavit* and observations. Finally, a unique identifier is provided for each specimen, which is then integrated into the herbarium collection.

### Step description

We reviewed the specimens from the collection and verified the coincidence with the assigned taxonomic information. Then, the information contained in the labels was extracted and digitised. To check taxonomy and accuracy, we compared scientific names with The Plant List (www.theplantlist.org) and the Taxon Match Tool (https://www.gbif.org/tools/species-lookup). Since the taxonomy of many groups has changed during the last decades, sometimes tricky to track, we also revised the Catalog of Chilean vascular plants (Rodriguez et al. 2018) to avoid any uncertainty about the identity of the species. Finally, the curated data were uploaded to the GBIF platform to make it publicly available (https://www.gbif.org/dataset/0f99f0f9-e32a-4deb-ad51-4e729dc9f274; Fonturbel et al. 2022).

## Geographic coverage

### Description

**Figure 1.**
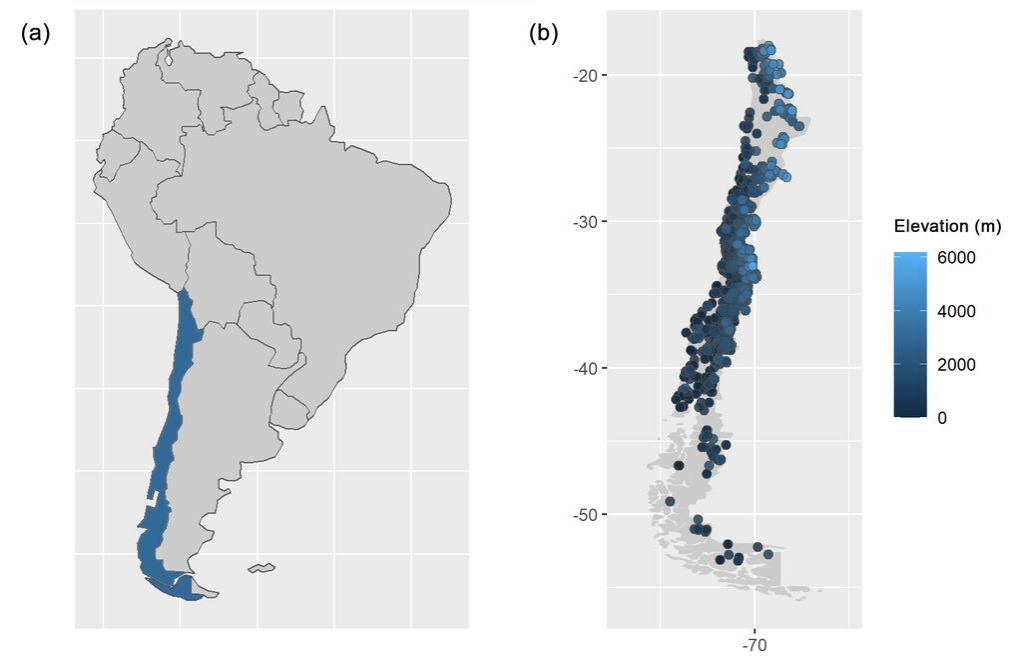
Location of **a** Chile within South America and **b** localities of collection of the specimens deposited in the PUCV Herbarium.

The majority of specimens were collected in Chile (95.6%; 10,244 specimens; Fig. 1), with the most represented administrative region being Valparaíso, where the Herbarium is based (53.1% of the total; 5,440 specimens), followed by the regions of Los Lagos (9.2%; 939) and Coquimbo (7.4%; 753). The Herbarium also contains some specimens from other South American countries, such as Argentina (359 specimens), Brazil (20), Paraguay (20), Bolivia (10) and Perú (4). North America and Europe are also represented by specimens from the USA (14), Germany (14), Austria (5) and Switzerland (1).

## Taxonomic coverage

### Description

**Figure 2.**
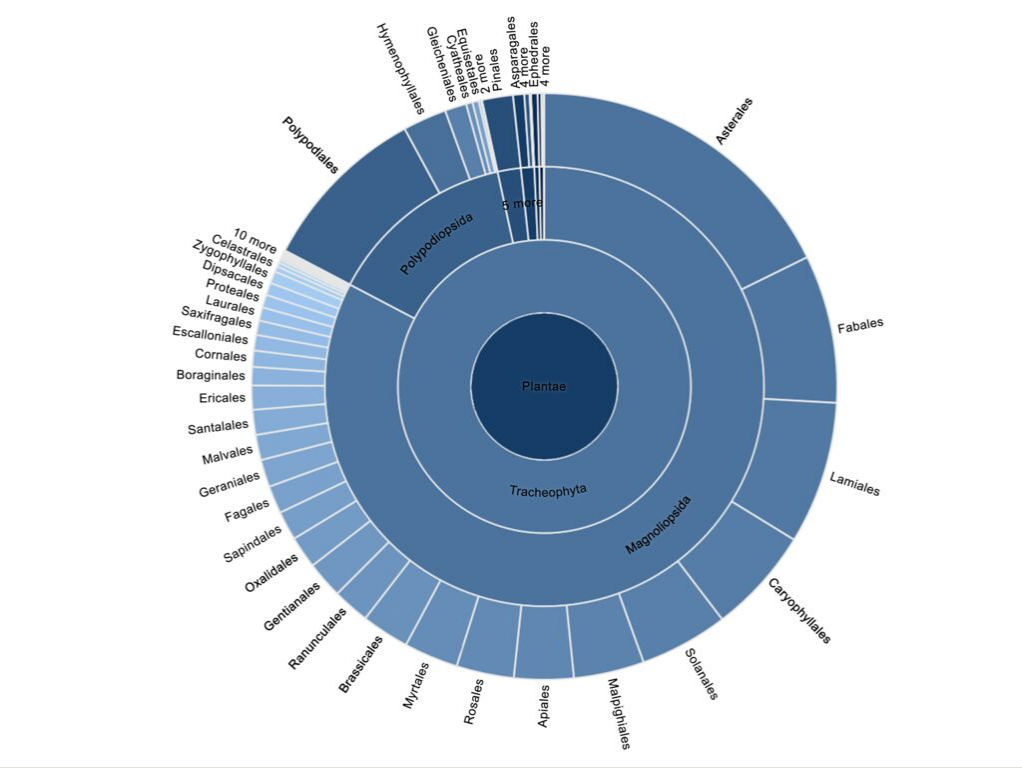
The general taxonomic distribution of occurrences.

Acanthaceae (1 specimen/1 name); Adiantaceae (30/3); Aextoxicaceae (9/1); Aizoaceae (22/9); Alstroemeriaceae (2/2); Altingiaceae (1/1); Amaranthaceae (125/48); Amaryllidaceae (27/11); Anacardiaceae (88/12); Annonaceae (4/2); Apiaceae (340/85); Apocynaceae (88/14); Aquifoliaceae (4/1); Araliaceae (6/3); Araucariaceae (10/3); Asparagaceae (8/3); Asphodelaceae (1/1); Aspleniaceae (141/8); Asteraceae (1889/540); Atherospermataceae (4/2); Begoniaceae (1/1); Berberidaceae (61/11); Betulaceae (7/3); Bignoniaceae (26/9); Blechnaceae (142/13); Boraginaceae (53/19); Brassicaceae (216/69); Cactaceae (10/9); Calceolariaceae (176/41); Campanulaceae (2/2); Cannabaceae (7/5); Caprifoliaceae (74/26); Cardiopteridaceae (20/1); Caryophyllaceae (120/30); Casuarinaceae (15/2); Celastraceae (32/5); Cephalotaxaceae (3/2); Ceratophyllaceae (1/1); Cervantesiaceae (1/1); Columelliaceae (4/1); Combretaceae (3/2); Convolvulaceae (77/22); Coriariaceae (5/1); Cornaceae (1/1); Crassulaceae (6/3); Cucurbitaceae (13/7); Cunoniaceae (32/4); Cupressaceae (94/24); Cyatheaceae (3/1); Cyperaceae (2/2); Cystopteridaceae (42/1); Dennstaedtiaceae (42/3); Dicksoniaceae (35/2); Dioscoreaceae (3/3); Dryopteridaceae (113/13); Ehretiaceae (9/5); Elaeagnaceae (3/2); Elaeocarpaceae (38/3); Ephedraceae (39/6); Equisetaceae (37/2); Ericaceae (54/17); Erythroxylaceae (2/2); Escalloniaceae (92/16); Euphorbiaceae (162/36); Fabaceae (812/241); Fagaceae (29/7); Francoaceae (12/2); Frankeniaceae (23/4); Gentianaceae (22/7); Geraniaceae (79/19); Gesneriaceae (13/3); Ginkgoaceae (3/1); Gleicheniaceae (128/4); Gomortegaceae (1/1); Goodeniaceae (10/1); Griseliniaceae (5/2); Grossulariaceae (37/10); Gunneraceae (8/2); Haloragaceae (36/1); Hydrangeaceae (9/3); Hydroleaceae (2/1); Hydrophyllaceae (47/7); Hymenophyllaceae (259/15); Hypericaceae (4/2); Iridaceae (3/3); Isoetaceae (2/1); Juglandaceae (2/1); Juncaceae (3/3); Krameriaceae (11/2); Lamiaceae (172/39); Lardizabalaceae (6/2); Lauraceae (59/9); Lentibulariaceae (4/1); Linaceae (30/4); Lindsaeaceae (12/1); Loasaceae (86/24); Loranthaceae (49/7); Lycopodiaceae (22/1); Lythraceae (35/11); Magnoliaceae (5/3); Malesherbiaceae (48/14); Malpighiaceae (18/5); Malvaceae (141/67); Marantaceae (1/1); Meliaceae (9/5); Misodendraceae (31/3); Molluginaceae (2/1); Monimiaceae (15/1); Montiaceae (74/24); Moraceae (13/7); Muntingiaceae (1/1); Myrtaceae (192/45); Nanodeaceae (1/1); Nephrolepidaceae (2/1); Nothofagaceae (111/9); Nyctaginaceae (30/9); Oleaceae (17/13); Onagraceae (91/21); Ophioglossaceae (3/1); Orchidaceae (27/7); Orobanchaceae (41/16); Oxalidaceae (120/29); Papaveraceae (77/12); Passifloraceae (11/7); Phrymaceae (42/8); Phytolaccaceae (30/6); Pinaceae (33/19); Piperaceae (7/4); Pittosporaceae (1/1); Plantaginaceae (132/37); Platanaceae (7/2); Plumbaginaceae (34/5); Poaceae (26/26); Podocarpaceae (43/7); Polemoniaceae (44/14); Polygalaceae (48/12); Polygonaceae (156/38); Polypodiaceae (87/7); Primulaceae (30/10); Proteaceae (69/7); Pteridaceae (371/23); Quillajaceae (15/1); Ranunculaceae (73/27); Rhamnaceae (109/21); Rosaceae (166/47); Rubiaceae (108/38); Rutaceae (23/11); Salicaceae (88/18); Salviniaceae (14/2); Santalaceae (7/1); Sapindaceae (50/10); Sapotaceae (14/4); Saxifragaceae (5/5); Schizaeaceae (3/1); Schoepfiaceae (58/6); Scrophulariaceae (37/9); Selaginellaceae (12/1); Simaroubaceae (2/1); Smilacaceae (1/1); Solanaceae (445/109); Strelitziaceae (1/1); Stylidiaceae (1/1); Tamaricaceae (2/1); Tectariaceae (4/1); Thelypteridaceae (21/2); Thymelaeaceae (7/1); Tropaeolaceae (67/8); Ulmaceae (4/3); Urticaceae (38/16); Verbenaceae (182/43); Violaceae (54/26); Viscaceae (1/1); Vitaceae (9/1); Vivianiaceae (71/8); Winteraceae (13/1); Woodsiaceae (2/1); Zygophyllaceae (60/7). The general taxonomic distribution of occurrences, including higher taxonomic categories, is presented in Fig. 2

## Temporal coverage

### Notes

**Figure 3.**
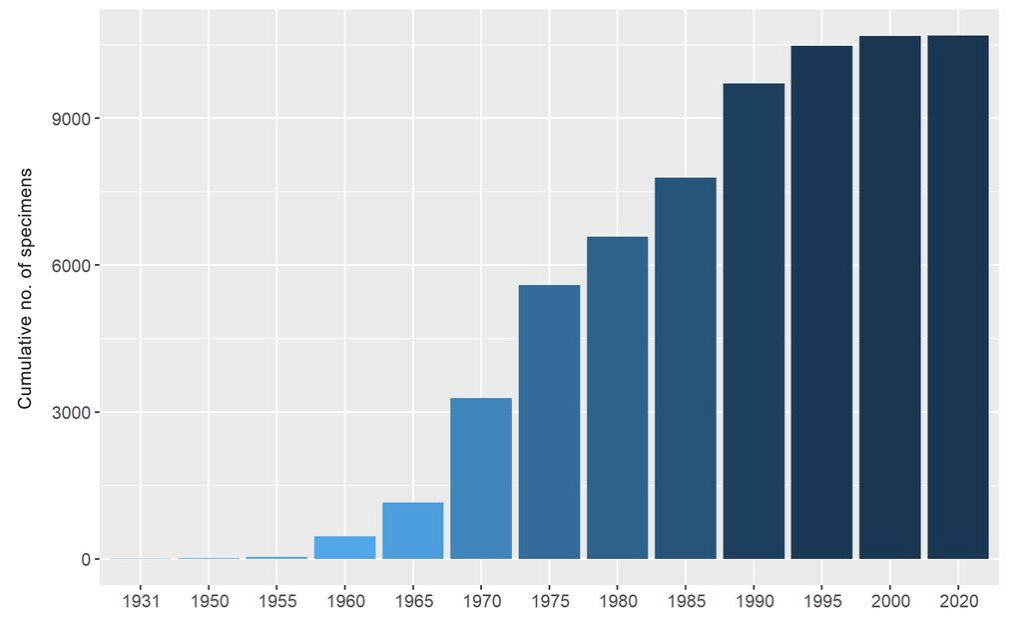
Temporal coverage of the specimens deposited in the PUCV Herbarium since the presumed Herbarium foundation in 1931.

Although the founding year of the PUCV Herbarium is unknown, it is presumed to have been in 1931 since the institution was founded in 1924 and the first specimen was deposited seven years later by the botanist Gualterio Looser. However, the Herbarium contains a few earlier records from 1900, probably integrated from the personal collection of the professors Otto Zöllner and Beatriz Palma. During the following years, few specimens were deposited until 1955, when the additions increased and were regularised (Fig. 3), mainly by the botanists Otto Zöllner, Bernardo Parra, Hugo Gunckel and Otto Magen.

## Collection data

### Collection name

PUCV Herbarium

### Collection identifier

PUCV

### Specimen preservation method

Dried and pressed

## Usage licence

### Usage licence

Creative Commons Public Domain Waiver (CC-Zero)

## Data resources

### Data package title

Vascular flora from the PUCV Herbarium

### Resource link

https://www.gbif.org/dataset/0f99f0f9-e32a-4deb-ad51-4e729dc9f274

### Number of data sets

1

### Data set 1.

#### Data set name

Vascular flora from the PUCV Herbarium

#### Download URL

https://www.gbif.org/dataset/0f99f0f9-e32a-4deb-ad51-4e729dc9f274

#### Description

This database includes 10,721 specimens from vascular flora (angiosperms, gymnosperms and pteridophytes) deposited in the Herbarium of the Pontificia Universidad Católica de Valparaíso (PUCV) in Chile. This botanical collection was started by the renowned botanist Otto Zöllner and represents a major natural historical legacy for central Chile, with decades of information represented through preserved specimens. This collection is currently deposited in the Curauma campus of the PUCV. This digitisation process is part of the PUCV effort to mobilise its biological collections and make them freely available through GBIF, encouraging national and international researchers to generate new knowledge, based on this invaluable heritage, which is a silent witness of the vast plant diversity that once existed in Chile and that is now vanishing due to anthropogenic drivers.

**Data set 1.** 

Column label	Column description
occurrenceID	The unique identifier of the occurrence.
basisOfRecord	The specific nature of the data record.
type	The nature of the resource.
eventDate	The date of collection.
year	Year of collection.
month	Month of collection.
day	Day of collection.
eventRemarks	Comments or notes about the Event.
scientificName	The full scientific name of the species.
scientificNameAuthorship	The authorship information for the scientific name.
verbatimScientificName	The scientific name of the species.
kingdom	The scientific name of the kingdom in which the taxon is classified.
phyllum	The scientific name of the phyllum in which the taxon is classified.
class	The scientific name of the class in which the taxon is classified.
order	The scientific name of the order in which the taxon is classified.
family	The scientific name of the family in which the taxon is classified.
genus	The scientific name of the genus in which the taxon is classified.
specificEpithet	The specific epithet of the scientific name.
infraspecificEpithet	The infrageneric part of a binomial name at ranks above species, but below genus.
taxonRank	The taxonomic rank of the most specific name provided in the scientificName.
identifiedBy	A list of names of people, groups or organisations who assigned the Taxon to the subject.
decimalLatitude	The geographic latitude of the geographic centre of a location, expressed in decimal degrees. Positive and negative values indicate north and south of the Equator, respectively.
decimalLongitude	The geographic longitud of the geographic centre of a location, expressed in decimal degrees. Positive and negative values indicate north and south of the Equator, respectively.
geodeticDatum	The ellipsoid, geodetic datum or spatial reference system (SRS) upon which the geographic coordinates given in decimalLatitude and decimalLongitude are based.
continent	The name of the continent in which the location occurs.
country	The name of the country in which the location occurs.
countryCode	The code for the country in which the location occurs according to ISO 3166-1-alpha-2 country codes.
stateProvince	The name of the next smaller administrative region than country in which the Location occurs.
locality	The specific description of the location.
maximumElevationInMetres	Maximum elevation to above sea level, expressed in metres.
language	Language in which the data and metadata are presented.
institutionID	The name of the institution having custody of the object(s) or information referred to in the record.
institutionCode	The acronym of the institution having custody of the object(s) or information referred to in the record.
catalogNumber	The unique code that identifies the record within the collection.
collectionCode	The name identifying the collection from which the record was derived.
recordedBy	A list of the person, people, groups or organisations responsible for recording the original Occurrence.

## References

[B7981177] Armesto Juan J., Manuschevich Daniela, Mora Alejandra, Smith-Ramirez Cecilia, Rozzi Ricardo, Abarzúa Ana M., Marquet Pablo A. (2010). From the Holocene to the Anthropocene: A historical framework for land cover change in southwestern South America in the past 15,000 years. Land Use Policy.

[B7954586] Bridson D., Forman L. (2000). The herbarium handbook.

[B8152637] Castro Sergio A., Figueroa Javier A., Muñoz-Schick Mélica, Jaksic Fabian M. (2005). Minimum residence time, biogeographical origin, and life cycle as determinants of the geographical extent of naturalized plants in continental Chile. Diversity and Distributions.

[B8025755] Fonturbel F, Lopez-Aliste M, Cordero S, Gálvez F (2022). Vascular flora from the PUCV Herbarium. Pontificia Universidad Católica de Valparaíso. Occurrence dataset.

[B8152628] Fuentes Nicol, Ugarte Eduardo, Kühn Ingolf, Klotz Stefan (2007). Alien plants in Chile: inferring invasion periods from herbarium records. Biological Invasions.

[B8152618] Fuentes Nicol, Pauchard Aníbal, Sánchez Paulina, Esquivel Jocelyn, Marticorena Alicia (2012). A new comprehensive database of alien plant species in Chile based on herbarium records. Biological Invasions.

[B8152594] Lendemer James, Thiers Barbara, Monfils Anna K, Zaspel Jennifer, Ellwood Elizabeth R, Bentley Andrew, LeVan Katherine, Bates John, Jennings David, Contreras Dori, Lagomarsino Laura, Mabee Paula, Ford Linda S, Guralnick Robert, Gropp Robert E, Revelez Marcy, Cobb Neil, Seltmann Katja, Aime M Catherine (2019). The Extended Specimen Network: A Strategy to Enhance US Biodiversity Collections, Promote Research and Education. BioScience.

[B7981110] Letelier Luis, Gaete-Eastman Carlos, Peñailillo Patricio, Moya-León María A., Herrera Raúl (2020). Southern species from the biodiversity hotspot of Central Chile: A source of color, aroma, and metabolites for global agriculture and food industry in a scenario of climate change. Frontiers in Plant Science.

[B7981168] Medel R., González-Browne C., Fontúrbel F. E. (2018). Pollination in the Chilean Mediterranean-type ecosystem: a review of current advances and pending tasks. Plant Biology.

[B7981189] Miranda Alejandro, Altamirano Adison, Cayuela Luis, Lara Antonio, González Mauro (2016). Native forest loss in the Chilean biodiversity hotspot: revealing the evidence. Regional Environmental Change.

[B7981131] Mittermeier Russell A., Turner Will R., Larsen Frank W., Brooks Thomas M., Gascon Claude (2011). Global biodiversity conservation: The critical role of hotspots. Biodiversity Hotspots.

[B7954700] Rodriguez Roberto, Marticorena Clodomiro, Alarcón Diego, Baeza Carlos, Cavieres Lohengrin, Finot Víctor L., Fuentes Nicol, Kiessling Andrea, Mihoc Maritza, Pauchard Aníbal, Ruiz Eduardo, Sanchez Paulina, Marticorena Alicia (2018). Catálogo de las plantas vasculares de Chile. Gayana. Botánica.

[B7980773] Teixeira-Costa L., Heberling J. M., Wilson C. A., Davis C. C. (2022). Parasitic flowering plant collections embody the extended specimen. Methods in Ecology and Evolution.

[B7981218] Wilkinson Mark D., Dumontier Michel, Aalbersberg IJsbrand Jan, Appleton Gabrielle, Axton Myles, Baak Arie, Blomberg Niklas, Boiten Jan-Willem, da Silva Santos Luiz Bonino, Bourne Philip E., Bouwman Jildau, Brookes Anthony J., Clark Tim, Crosas Mercè, Dillo Ingrid, Dumon Olivier, Edmunds Scott, Evelo Chris T., Finkers Richard, Gonzalez-Beltran Alejandra, Gray Alasdair J. G., Groth Paul, Goble Carole, Grethe Jeffrey S., Heringa Jaap, ’t Hoen Peter A. C, Hooft Rob, Kuhn Tobias, Kok Ruben, Kok Joost, Lusher Scott J., Martone Maryann E., Mons Albert, Packer Abel L., Persson Bengt, Rocca-Serra Philippe, Roos Marco, van Schaik Rene, Sansone Susanna-Assunta, Schultes Erik, Sengstag Thierry, Slater Ted, Strawn George, Swertz Morris A., Thompson Mark, van der Lei Johan, van Mulligen Erik, Velterop Jan, Waagmeester Andra, Wittenburg Peter, Wolstencroft Katherine, Zhao Jun, Mons Barend (2016). The FAIR guiding principles for scientific data management and stewardship. Scientific Data.

